# Recent Advances in the Use of Sourdough Biotechnology in Pasta Making

**DOI:** 10.3390/foods8040129

**Published:** 2019-04-18

**Authors:** Marco Montemurro, Rossana Coda, Carlo Giuseppe Rizzello

**Affiliations:** 1 Department of Soil, Plant, and Food Science, University of Bari Aldo Moro, 70126 Bari, Italy; carlogiuseppe.rizzello@uniba.it; 2 Department of Food and Nutrition, Helsinki Institute of Sustainability Science, University of Helsinki, 00100 Helsinki, Finland; rossana.coda@helsinki.fi

**Keywords:** pasta, fermentation, legume flours, sourdough, lactic acid bacteria

## Abstract

The growing consumers’ request for foods with well-balanced nutritional profile and functional properties promotes research on innovation in pasta making. As a staple food and a common component of diet, pasta can be considered as a vector of dietary fiber, vegetable proteins, vitamins, minerals, and functional compounds. The conventional process for pasta production does not include a fermentation step. However, novel recipes including sourdough-fermented ingredients have been recently proposed, aiming at enhancing the nutritional and functional properties of this product and at enriching commercial offerings with products with new sensorial profiles. The use of sourdough for pasta fortification has been investigated under several aspects, including fortification in vitamin B, the reduction of starch digestibility, and gluten content. Sourdough fermentation has also been successfully applied to non-conventional flours, (e.g., from pseudocereals and legumes), in which an overall increase of the nutritional value and health-promoting compounds, such as a significant decrease of antinutritional factors, were observed. Fermented non-conventional flours, obtained through spontaneous fermentation or using selected starters, have been proposed as pasta ingredients. As the result of wheat replacement, modification in textural properties of pasta may occur. Nonetheless, fermentation represents an efficient tool in improving, besides nutritional and functional profile, the sensory and technological features of fortified pasta.

## 1. Sourdough Fermentation: Innovations from the Past

Sourdough fermentation is one of the oldest biotechnologies widely employed in food production, as it converts cereal flour into attractive, palatable, and digestible products. Sourdough is a leavening agent traditionally obtained from a mixture of flour and water and spontaneously fermented by a complex microbiota dominated by lactic acid bacteria (LAB) and yeast populations. Many of the peculiar characteristics of sourdough and sourdough-derived foods are related to its microbiota metabolic activities. Particularly, like in many other fermented foods and beverages, the group of LAB plays a central role in sourdough production [1]. Overall, fermentation by LAB is considered a natural, sustainable, and effective tool for ensuring proper hygiene, rheology, sensory, and shelf life features, while simultaneously improving the functional/nutritional value of the food matrix [2]. The effects of sourdough fermentation are not only related to organic acids synthesis: the activation of the endogenous enzymes of the flour as well as the synthesis of microbial secondary metabolites, contribute to relevant changes of the fermented matrix, and affect the properties of the final products [3]. Among the effects conferred, the increase of the in vitro protein digestibility and amount of soluble fiber, and the decrease of the glycemic index, phytate content, trypsin inhibitors, and other anti-nutritional factors (ANF), have been observed, depending on the substrate used [4,5,6]. Besides the conventional employment of sourdough, innovative biotechnological protocols, based on the use of selected starters, different grain matrices, automatized bio-reactors, and semi-liquid formulations have been proposed in the last 15 years to extend its large-scale application in food industry. Sourdough biotechnology is considered a promising answer to modern consumers’ demand for natural foods with improved nutritional and functional value; thus, the extension of sourdough application to products different from leavened baked goods and widely consumed like pasta, represents a new opportunity for the food industry.

### 1.1. The Key Role of Lactic Acid Bacteria

Sourdough biotechnology has been widely studied in the last 15 years, in many of its aspects [7,8]. The more recent advances in the management of sourdough-related processes have led to classifying sourdough into three different types (I, II, and III) [9], mainly based on the protocols of propagation. Type I sourdough defines the traditional processes, characterized by daily propagation (back-slopping procedure), based on the use of the dough from the previous fermentation cycle as the natural starter for a new fermentation process [10]. Type II sourdough is usually a liquid/semiliquid dough fermented for a long time (up to 5 days), while type III mainly corresponds to a dried sourdough used as a flavoring agent [9,11]. In industrial conditions, commonly characterizing type II sourdough production, the use of selected starter cultures has been introduced, since it allows obtaining standardized quality products and it is less laborious. Several scientific studies on sourdough fermentation focused on LAB metabolic activities as the key factor in affecting the improvement of nutritional features [1]. During sourdough fermentation, LAB are mostly responsible of acidification and proteolysis, two phenomena widely affecting, at different levels, organoleptic, technological, and nutritional properties of sourdough, offering functional advantages achievable with its use. LAB cause rapid acidification of the raw material through the production of organic acids, mainly lactic and acetic acids, and contribute to the definition of sourdough complex profile through the synthesis of ethanol, aroma compounds, bacteriocins, exopolysaccharides, and several enzymes [12].

A trend that is increasingly attracting bakery industries as well as consumers is the use of non-conventional flours, including cereals different from wheat, legumes, and pseudo-cereals, for the production of novel products, characterized by peculiar flavor and high nutritional value (e.g., abundance of proteins with high nutritional value, dietary fibers, polyphenols, minerals). The challenge in fermenting such matrices is represented by the necessity to combine good technology and sensory properties with nutritional/health benefits [7,13]. The LAB microbiota of several cereals, legumes, and pseudo-cereals have been deeply investigated with the aim of studying biodiversity and finding suitable starter cultures for sourdough fermentation. Indeed, the choice of starter cultures has a critical impact on the final quality of the product, and strains with good adaptation capacity can deliver optimal results in specific sourdough fermentations. It has been observed that lactic acid bacteria deriving from a certain matrix usually have a good performance in the same matrix where they have been isolated from [7,13,14].

In the following sections, the nutritional and functional features associated with sourdough fermentation are reviewed, aiming at introducing the advantages of the innovative application of this old biotechnology in pasta making. 

### 1.2. Nutritional Effects of Sourdough Fermentation

Nutritional characteristics are commonly correlated to the chemical composition of foods and its ingredients. The amount of carbohydrates, proteins, lipids, as well as minerals and vitamins are usually described for each food and communicated to the consumer through labels. Although directly correlated with the nutritional value of food, composition does not provide information about bioavailability of macro- and micro-nutrients and functional and ANF, whose presence is largely affected by processing (including fermentation steps).

The Glycemic Index (GI) corresponds to the incremental area under the glucose response curve after the ingestion of different portions of food with the same amount of carbohydrates [15]. This parameter is employed to classify foods, since it is correlated with the incidence of diet on the nutritional status and on the development of different pathologic conditions (obesity, diabetes, and cardiovascular disease). Sourdough fermentation can lower GI reducing starch digestibility due to the formation of organic acids and other complementary mechanisms developed by LAB metabolism [15]. 

As well as the effect on GI, the activity of LAB on the organic nitrogen fraction has been widely investigated [16,17]. Sourdough LAB have relevant proteolytic activity due to the cell-wall proteinase, intracellular peptidases, and specific membrane transporters [3] while concurrent acidification allows the activation of endogenous flour proteases [3]. Proteolysis during LAB fermentation leads to a progressive hydrolysis of native proteins and to the increase of peptides and free amino acids (FAA) concentrations. It was reported that the release of FAA fermentation allows to reduce salt addition to foods without affecting their sapidity [2]. Such an effect is of great interest considering that cereal-derived products are the main source of sodium chloride in Western diet, whose consumption has been associated with the risk of cardiovascular diseases [18,19].

Moreover, sourdough fermentation can be considered as a pre-digestion process driven by the enzymatic activity of LAB. As a consequence, higher digestibility of protein fraction and better nutritional indexes are commonly associated with sourdough-containing products [20]. Proteolysis is already used to decrease the amount of gluten, potentially causing allergy and intolerance responses in sensitive individuals [3], and to increase the digestibility of protein-rich flours (e.g., legumes) widely used to fortify cereal-based products [21]. Bioprocessing protocols have been set up to manufacture cereal-based foods with a reduced or fully hydrolyzed gluten content, making them safe for consumption by individuals with celiac disease [3]. 

LAB enzymatic activities can contribute to increase the amount of soluble fibers in food products. Fiber solubilization is correlated with flour endogenous and microbial enzymes, such as xylanases [22] that, acting on arabinoxylans, allow the solubilization of the fiber insoluble fraction. The increase of water-extractable arabinoxylans was correlated with the delay of carbohydrate digestion and absorption rates and to a decrease of the glycemic and insulinemic responses [23,24,25]. Moreover, the health benefits of soluble oligosaccharides, derived from hydrolysis of water-unextractable arabinoxylans, were correlated with prebiotic effect and anticarcinogenic, antioxidant, and hypocholesterolemic properties [26].

LAB can produce exopolysaccharides (EPS), compounds able to improve textural properties of wheat and gluten free baked goods [27]. Functional features such as immunomodulation, antioxidant, hypocholesterolemic, and prebiotic activities were also attributed to EPS [28]. 

It was demonstrated that sourdough fermentation could reduce the phytate content of flours. Phytic acid is very abundant in the outer layer of grains and it is considered the main cause for low mineral bioavailability of cereal flours, due to the formation of phytate-mineral complex. The fermentation process with lactic acid bacteria can efficiently degrade the phytate complex thanks to the activation of endogenous and microbial phytases [29].

### 1.3. Functional Effects of Sourdough Fermentation

The in situ synthesis of bioactive compounds during the fermentation process has been extensively studied. It was shown that sourdough fermentation led to the enrichment of different anti-hypertensive compounds. Among these, γ-aminobutyric acid (GABA) can be produced by LAB by glutamate decarboxylase, to counteract the acidification of the substrate [2]. This amino acid was shown to decrease blood pressure in moderately hypertensive patients with the dietary intake of 10–12 mg/day [30]. The production of GABA using sourdough biotechnology from legume and pseudocereal flours was also explored [4,31]. 

The generation of bioactive peptides during sourdough fermentation has been shown in several studies. Bioactive peptides, commonly represented by sequences of 3–20 amino acid units, can exert several physiological effects [32,33]. Among them, angiotensin I-converting enzyme (ACE) inhibitory peptides can lower blood pressure through vasodilator activity. The synthesis of these peptides was observed in wheat sourdoughs; moreover, the presence of the VAP (Valine-Alanine-Proline) epitope in such peptides, as previously found in anti-hypertensive sequences deriving from caseins, was observed [34].

Sourdough fermentation has also been correlated with the increase of antioxidant activity. This phenomenon is due to the synthesis of antioxidant peptides and to the increase of phenolic acids concentration, as a consequence of the release of encrypted peptides from native proteins and of the hydrolysis of complex phenolic compounds, respectively [2,35,36]. The antioxidant activity of fermented flour was demonstrated in different vegetable matrices, including cereals, pseudocereals, and legume flours [21,37,38,39].

Innovative sourdough-based biotechnologies have been set-up aiming at producing other bioactive compounds including anticancer/antiproliferative, hypocholesterolemic, anti-inflammatory, and immunomodulatory peptides [40].

## 2. Cereal-Based Sourdough in Pasta Making

Traditionally, the production of pasta is said to originate in China, but pasta was known many centuries before in the Mediterranean area. Etruscan people knew the technique of preparing noodles or lasagne, while the description of this food was found in many documents from the Greco-Roman age [41]. Over time, pasta became increasingly important in the South of Italy, thanks to the optimal climatic conditions for the durum wheat cultivation and for pasta drying. With the globalization of the agro-industrial culture, including the change of dietary habits, pasta became popular worldwide. Today, pasta represents a primary component of the diet in many countries [42].

The conventional process for pasta production does not include a fermentation step. Pasta is traditionally produced by a rapid extrusion of a durum wheat semolina dough through dies, followed by a drying stage in strictly controlled conditions (dried pasta).

With the aim of enhancing the nutritional and functional properties of pasta, as well as enriching the commercial offer with products with new sensorial profiles, different recipes including sourdough-fermented ingredients have been recently proposed. 

### 2.1. Nutritional Aspects

The use of refined wheat flour, although having high technological properties and stability, leads to the obtainment of food containing less of the beneficial compounds mostly present in germ and bran, like dietary fibers, vitamins, and polyphenols. Today, thanks to the increased consumers’ attention towards the nutritional properties of foods, the demand for food products with a well-balanced nutritional composition is increasing. For this reason, the use of sourdough for pasta fortification has been investigated in several ways, including the fortification in vitamin B, riboflavin, and the reduction of starch digestibility and GI.

In cereals, a large portion of vitamins is located in the germ and aleurone layer, which are removed during milling. In particular, B-vitamins are an essential component of human diets, as they support growth, erythrocyte formation, and the energy-producing metabolism. Riboflavin is a B-group vitamin, mostly present in the aleurone layer of wheat, it is stable to high temperature, high oxygen and acid content but unstable to alkali and light exposure [43,44]. Riboflavin deficiency is found in 6–15% of the worldwide population and persists in both developing and industrialized countries. Many microorganisms, including LAB, have been studied for their capability to produce riboflavin. The use of riboflavin producing LAB has emerged as a tool to produce fermented and fortified foods at the same time [45]. Recently, aiming at vitamin fortification of wheat flour to be used in pasta making, LAB strains were selected based on roseoflavin-resistance assay, riboflavin synthesis capability, and stability of riboflavin-overproducing phenotype [42]. Their use was proposed as an alternative way to increase riboflavin content in cereal-derived products, instead of the addition of the chemical synthesized one. In particular, riboflavin-overproducing *Lactobacillus plantarum* UNIFG1 and UNIFG2, isolated from sourdough, were used as starters for a pre-fermentation step of semolina and finely ground semolina (re-milled) [42]. The latter was the most suitable substrate for riboflavin fortification by LAB, probably because the re-milling process provides damaged starch, promoting LAB growth. Riboflavin content was monitored during all the steps of the pasta production process, including mixing, extrusion, and drying. The final concentration determined after cooking was ca. 2.48 μg/g. Taking into account 100 g of serving portion, fortified pasta can significantly contribute to riboflavin intake, considering values of 19.2% of the Reference Daily Intake (RDI) for men, and 22.7% of the RDI for women [46]. 

Low GI diets have been shown to protect against type II diabetes, cardiovascular disease, obesity, metabolic syndrome, and some types of cancer [47]. Since human diet is based on staple starch rich foods—such as rice, bread, and pasta—many attempts have been made to reduce starch digestibility, gelatinization, and hydrolysis rate or increase the undigestible starch fraction in foods, especially concerning the prevention of the above conditions in industrialized countries. Overall, pasta GI is lower than that of bread [48], and commonly ranges from 40 to 60 [48]. Nonetheless, common pasta portion sizes (i.e., 80–100 g) often provide relevant amount of carbohydrates in the diet [49], and GI reduction is considered one of the main objectives of the scientific and industrial research on pasta [50,51,52]. 

Sourdough fermentation strongly affects starch digestibility and GI of leavened baked goods [3], thus it is potentially applicable also in pasta making. The production of organic acids during sourdough fermentation has many positive effects on starch digestibility: lactic acid is responsible for low starch digestion, while acetic and propionic acids for the prolonged gastric emptying rate. 

In particular, it was hypothesized that lactic acid promotes the interaction between starch and gluten, thus limiting starch bioavailability [53]. This phenomenon is not solely due to the pH drop, but is correlated with the specific organic acid.

A recent study evaluated the starch digestibility in fresh pasta manufactured with semolina-based liquid sourdough fermented by *Saccharomyces cerevisiae* PCC1140 and *Lactobacillus alimentarius* PCC859. Sourdough fermentation did not affect the total starch content but induced several molecular changes. Compared to an unfermented control, sourdough led to the decrease of the slowly digestible fraction (43.0% vs. 49.8%) and to the increase of the not-digestible (40.2% vs. 34.5%) and retrograded starch fractions [54].

### 2.2. The Celiac Issue and Gluten Reduction

Gliadins and glutenins are the protein fractions of wheat responsible for the formation of gluten during the mixing of flour with water. Products made using wheat or other cereals, in which homologous proteins are found, like rye and barley, are associated with gluten related disorders [55]. These disorders (celiac disease, wheat allergy, and non-celiac gluten sensitivity) having an estimated global prevalence around 5%, could show similar clinical manifestations and are due to gluten assumption. Celiac disease and wheat allergy could be diagnosed based on a combination of the patient’s clinical history and specific tests, while non-celiac gluten sensitivity is still considered a diagnosis of exclusion, in the absence of clear-cut diagnostic criteria [55]. Gluten sensitivity symptoms disappear in people that follow a gluten free diet. Nevertheless, the threshold of gluten intake related to the adverse reaction in subjects affected by gluten sensitivity is still debated and it has not yet been precisely established [56]. People suffering from gluten sensitivity are commonly affected by irritable bowel syndrome (IBS), an intestinal disorder that causes abdominal pain, bloating, diarrhea, constipation, and gut microbiota unbalance [2]. Besides gluten, IBS is also related to the intake of other nutrients or ANFs such as lipopolysaccharides, amylase/trypsin inhibitors, wheat germ agglutinins (WGA), and fermentable oligo-, di-, and monosaccharides and polyols (FODMAPs) [57].

To date, the only treatment to avoid complications is a gluten-free diet. Europe and United States law set the limit to define gluten free products to 20 ppm [58,59]. This limit is also applied to ingredients and food where a gluten-containing grain or flour is used, but have been processed to remove gluten. 

Sourdough biotechnology was used to reduce gluten content of wheat flour under the limit of 20 ppm to produce gluten free pasta [60]. The process included the development of a semi-liquid dough having dough yield (DY, dough weight × 100/flour weight) of 220, which was fermented for 24 h at 37 °C with four selected LAB (*Lactobacillus alimentarius* 15M, *Lactobacillus brevis* 14G, *Lactobacillus sanfranciscensis* 7A, and *Lactobacillus hilgardii* 51B) previously isolated from sourdoughs [16,60]. These LAB were selected based on their ability to hydrolyze gliadin fractions and various proline-rich oligopeptides, including the 33-mer epitope, considered as the key-factor in determining gluten-related disorders [61]. After fermentation, the sourdough was freeze-dried and milled to be used as fermented ingredient in the final recipe of pasta. A 3:7 ratio of sourdough flour and buckwheat (*Fagopyrum esculentum*) flour, respectively, was used. Different tests were performed to define the degree of protein hydrolysis, focusing on gliadins degradation. In particular, a specific sandwich ELISA (Enzyme-Linked Immunosorbent Assay) based on the use of the R5 antibody, commonly used to identify immunoreactive proteins and peptides able to induce toxic responses in celiac patients, showed a gluten concentration five-folds lower in experimental fermented pasta compared to the unfermented control [60].

The sensory analysis revealed that the experimental pasta was less sticky and firm than pasta made without fermentation, without any other significant difference. 

In a follow up study, a process to manufacture pasta with a higher amount (50%) of wheat flour, rendered gluten-free through LAB fermentation, was developed [50]. The bioprocess consisted of fermentation of wheat flour by sourdough lactic acid bacteria (*Lactobacillus sanfranciscensis* 7A, LS3, LS10, LS19, LS23, LS38, and LS47, *Lactobacillus alimentarius* 15M, *Lactobacillus brevis* 14G, and *Lactobacillus hilgardii* 51B) previously selected based on their ability to hydrolyze Pro-rich peptides [50] in the presence of fungal proteases (from *Aspergillus oryzae* and *Aspergillus niger*). Wheat flour bioprocessing was carried out at 30 °C for 48 h. For pasta making, 50% of the above freeze-dried sourdough (DY of 500) and 50% of pre-gelatinized rice flour, calculated on the total amount of flour, were used. Fermentation led to an increase of total FAA and peptides in the water-/salt-soluble fraction, deriving from the gliadin and glutenin proteolysis. Particularly, the amount of amino acids was 15-folds higher than that found in the unfermented dough (0.987 ± 0.043 vs.15.212 ± 0.125 g/kg). The gluten content of fermented dough was lower than 10 ppm. The nutritional indexes of rendered gluten free wheat pasta were similar to those of commercial durum wheat pasta. The in vitro protein digestibility and the protein efficiency ratio (PER) were higher in the experimental sample. An acceptable structure of pasta made using sourdough was kept thanks to pre-gelatinized rice flour [62]. Moreover, fermentation led to the decrease of the starch hydrolysis rate and hydrolysis index (HI) to 58.8% [50]. The authors concluded that the use of wheat flour rendered gluten-free in pasta could be considered a good alternative to other gluten-free ingredients (rice or maize flours) usually employed in gluten-free formulations.

In a recent study, pasta with a reduced gluten content was produced using fermented semolina [56]. Gluten hydrolysis was carried out by sourdough fermentation using a pool of selected lactobacilli and fungal proteases [50]. Pasta made using the treated flour (containing 50% of the native gluten) had lower amount of Ca^++^ and did not show significant differences in FODMAPs content compared to the control, made with native wheat flour. Experimental pasta was tested in a randomized crossover-controlled trial on 20 IBS patients showing the positive effect of reduced gluten pasta after 2 weeks of daily consumption [56].

### 2.3. Textural and Cooking Properties and Sensory Profile

The main technological parameters of pasta made with unconventional and fermented ingredients are generally determined after cooking, since this process causes relevant changes in physicochemical characteristics of the product. 

Optimal cooking time (OCT, defined as the time needed for the disappearance of the white core) and cooking loss (corresponding to the amount of solid residues in cooking water), are considered as quality parameters for pasta. Shorter OCT and lower cooking loss for pasta made with fermented flour compared to the unfermented control were observed [54]. In the above study, a correlation between the use of sourdough and firmness increase was reported, thus hypothesizing a positive effect of organic acids in the interaction between starch and gluten, which contributed to obtaining a firmer network [54].

Nevertheless, several studies highlight the strict dependence of the textural properties from the amount of fermented flour included in the recipe as well as the fermentation parameters (e.g., fermentation time, degree of proteolysis). For example, when semolina was fermented aiming at decreasing gluten, the technological quality of the pasta became inferior, if compared to the unfermented control [50]. In particular, cooking loss increased and firmness decreased, due to the extensive proteolysis occurring during LAB fermentation, which weakened the gluten network.

The structure of pasta obtained with wheat flour rendered gluten-free by fermentation in combination with rice flour [61] was analyzed through epifluorescence/fluorescence microscopy. The analysis of the microstructure revealed the extensive protein degradation of the fermented wheat flour, and the presence of few and disorganized protein spots deriving exclusively from rice flour. The protein structure of pasta containing fermented wheat flour presented inhomogeneous aggregates, similarly to those commonly observed in commercial gluten free pasta (made with natural gluten-free ingredients), but completely different from the homogeneous reticular structure observed for conventional durum wheat pasta. The starch structure appeared similar in all samples analyzed. As expected, in conventional pasta the strong gluten network avoided starch swelling and deformation during cooking, phenomenon characterizing commercial and rendered gluten free pasta. In these cases, amylose leaked out of the granules. As the consequence of the microstructure organization, textural properties of pasta made with rendered gluten free flour were similar to those of commercial gluten free pasta (made with natural gluten free ingredients, mainly rice and maize flours). Nevertheless, hardness, gumminess, and chewiness, determined by instrumental texture profile analysis, were significantly higher in the experimental gluten free pasta compared to the commercial counterpart.

Overall, sensory analysis showed that pasta made with fermented flour significantly differed from conventional products. Clearly, many sensory properties were associated to the textural features. Several studies demonstrated that acidic smell and flavor derived from LAB fermentation were markedly attenuated by cooking, thanks to the high temperature and the solubilization of hydrophilic small compounds in water. Nevertheless, also in this case different results were observed on the basis of the amount of fermented flour included in the recipe and the related fermentation parameters.

When 30% of native wheat flour was substituted with freeze dried wheat flour previously fermented by a selected pool of LAB, lower scores for stickiness and firmness perception were found in comparison to the control, a pasta formulation made with the same recipe but including only unfermented ingredients. Overall, odor and flavor did not significantly differ between the two products, and the experimental pasta was positively judged [50].

No significant differences were found for perceived stickiness, presence of flaws and chewiness between a commercial gluten-free pasta and an experimental pasta obtained using wheat flour rendered gluten-free by fermentation [61], although an unusual taste characterized experimental pasta, probably due to the acidity derived from the fermented wheat flour component. However, the overall acceptance was not affected, even though the experimental pasta including fermented wheat flour presented the lowest lightness.

Besides the potential application in gluten degradation, sourdough fermentation was used to improve the quality of natural gluten-free ingredients. Among these, sorghum is considered an excellent source of proteins and antioxidant compounds [63]; it is also characterized by a lower amount of starch, compared to other cereals, which is of interest for diabetic or obese people [64]. A dried sorghum sourdough (DY of 300) was employed as pasta ingredient [65]. In particular, the fermented sorghum was mixed with parboiled brown rice and pre-gelatinized rice flour at 15% w/w in the final recipe. The addition improved pasta cooking quality compared to the control containing unfermented sorghum, with lower firmness and cooking losses, and higher water absorption [66]. The low cooking loss reflected a suitable protein linkage, positively contributing to a stable network in the final product [65].

Table 1 lists, not exhaustively, the characteristics of the experimental pasta made using the fermented cereals flour described before.

## 3. Fermented Legumes and Pseudocereals in Pasta Making

The use of flours different from wheat as wheat replacers is currently considered by food industry as a successful strategy to meet the demand of healthy and alternative products from an increasing niche of consumers, also due to their positive image and association with natural and traditional ethnic foods [13]. Legumes and pseudocereals—such as quinoa, amaranth, and buckwheat—have very different chemical composition and technological properties compared to wheat. 

Quinoa (*Chenopodium quinoa* L) is a pseudo-cereal with high-protein content and balanced amount of essential amino acids, especially due to the high ratio of histidine and lysine, in comparison with cereal proteins. Lipids in quinoa are rich in unsaturated fatty acids and are characterized by a balanced linoleic:linolenic acid ratio [67]. Quinoa flour is rich in dietary fiber and bioactive compounds, and the use of its flour as food ingredient is largely appreciated by consumers. In recent years, the production of quinoa markedly increased over the world, thanks to the large adaptability of this crop to different climates and soils [68]. Faba bean (*Vicia faba* L.) flour is obtained from the dehulled seeds, is considered a source of the amino acids of which cereal based products are lacking (i.e., lysine and threonine). Faba bean is cultivated in many parts of the world, its employment in food and feed industry is increasing and it can be considered as a potential soy substitute. Unfortunately, faba bean seeds contain antinutritional compounds—including condensed tannins, α-galactosides, pyrimidine glycosides, and trypsin inhibitors—which limit its utilization. Aiming at removing them, fermentation with LAB was successfully applied [51].

Similarly to faba bean, pigeon pea or red gram (*Cajanus cajan*) is considered a valuable source of proteins, minerals, and vitamins, although it is also rich in antinutritional compounds such as phytic acid, polyphenols, saponins, trypsin inhibitors, and oligosaccharides. In this legume the amount of lysine is high while the sulfur amino acids, methionine and cysteine, are lacking 

Regardless the biotechnology used for the production, pasta containing legumes and pseudocereals had higher content of proteins and fibers and lower starch content compared to the conventional one. Nonetheless, fermentation contributed to improving, not only the nutritional profile, but also the technological features of fortified pasta. Lactic acid bacteria fermentation of pseudocereals and legumes flours has determined an overall enhancement of the nutritional value by increasing health-promoting compounds and decreasing the ANF [69,70,71,72,73].

When used for pasta making, fermented flours , obtained through spontaneous fermentation or using selected starter strains, are freeze-dried and added to the final recipe in percentages varying from 10% to 100% [52].

### 3.1. Effect on Chemical, Nutritional, and Functional Quality

Faba bean (*Vicia faba*) flour fermented by *Lactobacillus plantarum* DPPMAB24W was used in different amounts (i.e., 10, 30, and 50%) to substitute semolina [51]. Overall, it was reported that LAB fermentation of faba bean flour led to the degradation of ANFs such as trypsin inhibitors, condensed tannins [74,75], and vicine and convicine [76], pyrimidine glycosides causing favism in susceptible individuals.

**Figure 1:**
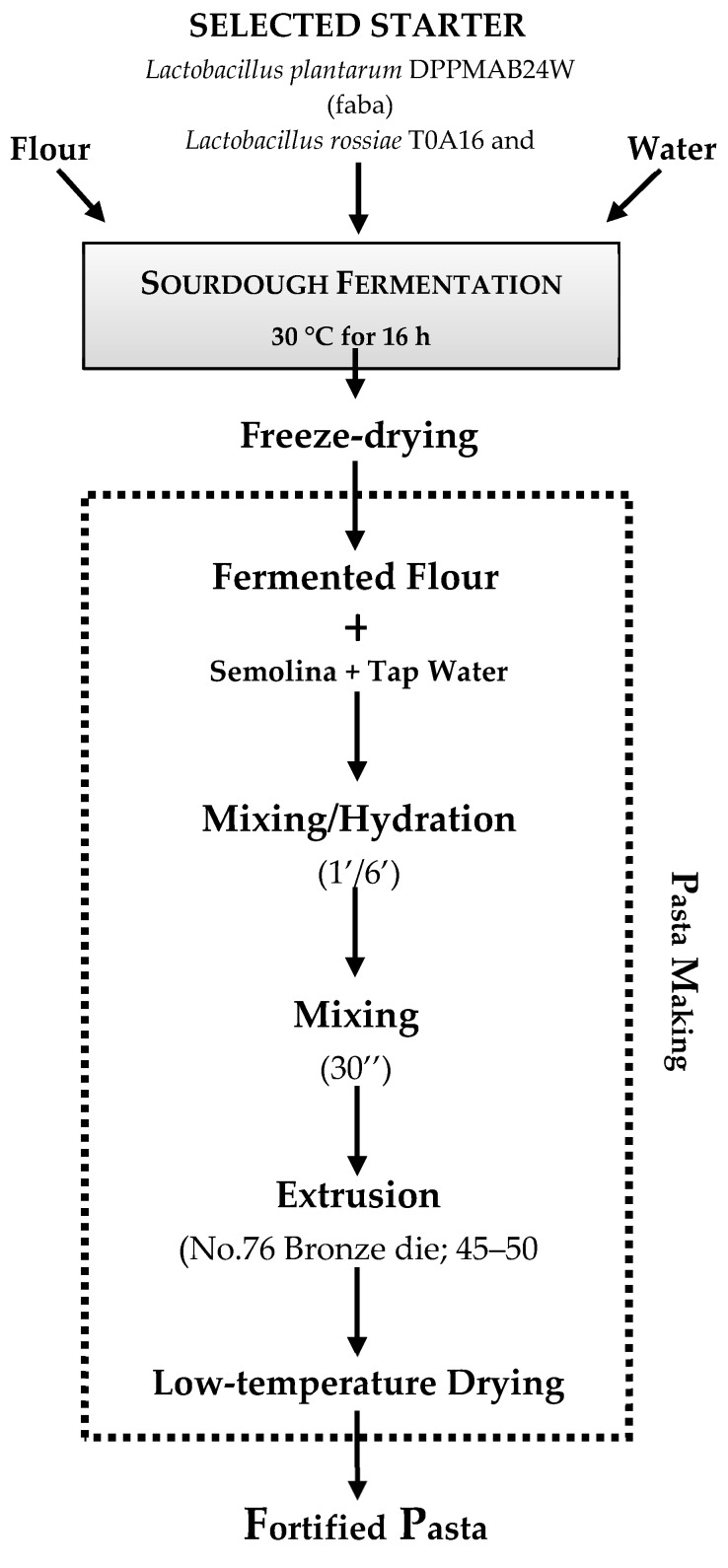
Example of the production process of pasta fortified with faba bean [51] or quinoa [80] flours fermented with selected lactic acid bacteria.

A faba bean semiliquid sourdough (DY of 160) was freeze dried before the inclusion into the final recipe of pasta (Figure 1). Protein and dietary fiber content in fortified pasta increased in direct proportion to the percentage of semolina replacement with both raw or fermented faba bean flours. However, a higher content of peptides and FAA was observed in pasta containing fermented faba bean, as a consequence of the proteolysis during fermentation. Faba bean flour had higher resistant starch (RS) content than semolina flour; which, upon fermentation, further increased due to the biological acidification. Proteolysis phenomena during faba bean fermentation also led to the increase of protein digestibility. Essential amino acids index (EAAI) and biological value (BV), corresponding to the ratio of essential amino acids of the sample proteins and the protein amount potentially retained by the human body after ingestion, respectively, were markedly higher at the substitution level of 30% and 50% of fermented faba bean flour, compared to semolina pasta and to pasta obtained with the same amount of unfermented faba bean flour. Experimental pasta also had the highest Protein Efficiency Ratio (PER) index, which reflects the capacity of a protein to support the body weight gain [51]. Fermented faba bean flour addition also affected the Nutritional Index (NI), a parameter considered as a global predictor of the protein quality of foods [77]. This index was two folds higher in pasta fortified with 30% of fermented faba. Nevertheless, when 50% replacement of the semolina was used, a decrease of the NI was observed, as a consequence of the gluten decrease that causes a weak microstructure of the pasta, unable to retain the soluble protein fraction [51]. The fortification of pasta with fermented faba bean flour led to the reduction of HI and, consequently, of the GI [51,74,78], thanks to the high concentration of dietary fibers and RS, and to the biological acidification, one of the main factor for decreased starch hydrolysis rate and GI [79].

Fermented faba bean flour was also used as the sole ingredient for pasta production [52]. In this case, LAB fermentation lasted 48 h at 30 °C and fermented dough was freeze dried before use in pasta making. Fermented and unfermented faba bean pasta showed similar protein (35.4% and 35.3%, respectively) and starch content (43.5% and 43.3%, respectively), with the protein being significantly higher (14.2%) and the starch significantly lower (73.5%) than semolina pasta. Conversely, RS was found higher in fermented compared to unfermented faba bean pasta. The use fermented faba bean flour was suggested as means to decrease the GI of commercial gluten free products [55], usually higher than that of conventional foods [48]. 

Quinoa flour fermented by *Lactobacillus rossiae* T0A16 and *Lactobacillus plantarum* T6B10 was used at 20% substitution level for pasta making [80]. The two starters were previously isolated from quinoa fermented flour and selected based on acidification capability and proteolytic activity. An increase in protein (+20%) and dietary fibers (+49%) was found in fermented quinoa enriched pasta compared to the standard semolina control. Starch content decreased in fortified pasta independently on the fermentation. A progressive increase in peptides (2.7 and 7.1 vs. 1.9, mg/g of pasta) and FAA (0.33 and 0.72 vs. 0.23, mg/g of pasta) was observed in pasta fortified with raw or fermented quinoa compared to the control. In addition, a higher total phenol content was found. The fortification with fermented quinoa flour increased all nutritional indexes and significantly decreased the HI of pasta to 52.7% [80].

Spontaneously fermented and acidified pigeon pea (*Cajanus cajan*) (presumably due to LAB growth), was also used in pasta making [69,70]. Significantly higher amounts of lysine, threonine, asparagine and arginine and lower levels of glutamine and proline were observed in pasta supplemented with 10% of fermented pigeon pea flour compared to semolina control [81]. Overall, fermented pigeon pea flour addition had a positive effect on protein quality, as determined by the Chemical Score (CS) index, that resulted markedly higher in fortified pasta.

After cooking, fortified pasta exhibited higher asparagine, glycine, lysine and threonine concentration compared to semolina pasta. In particular, the increase of lysine content is a remarkable outcome, since lysine markedly contributes to improve the biological value of cooked pasta, being the most limiting amino acid of wheat-derived foods [7,52,80,82]. Compared to semolina pasta, true protein digestibility (TD) and PER markedly improved (6 and 73%, respectively) in pasta fortified with fermented pigeon pea as consequence of the complementarity of amino acids composition deriving from legumes and cereal proteins [70,81]. Examples of production processes for making pasta fortified with non-conventional fermented flours are schematized in Figure 1.

### 3.2. Sensory Acceptability and Textural Properties of Fortified Products

The obtainment of good sensory and textural properties represents the main challenge in the use of fermented ingredients in pasta making. Like structural characteristics, the study of the sensory properties of fortified pasta can be considered as a predictive tool to investigate the consumer perception and acceptability, since it provides useful information for the design of novel commercial foods.

Literature has shown several differences in sensorial attributes and textural properties between pasta fortified with pre-fermented ingredients and the conventional one. 

As expected, unusual flavors were perceived by trained assessors when pasta included relevant aliquots (up to 50%) of pre-fermented wheat flour [61].

Fermentation showed an important role in the improvement of sensory and textural characteristics of legume flours, since it allowed the elimination of beany flavor [83]. Nevertheless, when non-wheat fermented flours were used for fortification, replacement percentages lower than 50% were overall suggested. Increased chewiness, sourness, flavor, and off-flavor intensity were observed when fermented faba bean was added to pasta [52], such as the onset of the red color, as the consequence of Maillard reaction [84]. Moreover, pungent odor and flavor, and aftertaste perception characterized pasta containing 30% of fermented faba flour [51].

A 10% level of semolina replacement with fermented pigeon pea flour was reported as the maximum level to avoid loss of sensorial acceptability [70], since a further increase of only 2% of fermented flour was judged by panelists as not pleasant. Besides the nutritional advantages previously described, the addition of 20% of fermented quinoa flour to pasta conferred higher intensity of flavor and taste compared to control [80]. Compared to the use of native non-wheat flours, it was found that LAB fermentation improved elasticity and cohesiveness of fortified quinoa pasta, probably due to the moderate proteolysis occurring during fermentation [51].

Texture instrumental analysis of cooked pasta provides information on the structural parameters that are strictly correlated with sensory perceptions [52]. Such evaluation also allows an accurate comparison between fortified and conventional semolina pasta based on the physical response to mechanical stress. For example, texture profile analysis (TPA) applied to pasta made with 100% fermented faba bean flour showed high gumminess and chewiness and decreased elasticity compared to a not-fermented faba bean pasta [52], while pasta made with fermented quinoa showed the increase of overall elasticity (resilience and cohesiveness) and tenacity (hardness and fracturability) parameters compared to a conventional semolina control [80].

Microscopic structure of pasta containing faba bean flour was evaluated in gluten free [52] and wheat-based [51] formulations. Pasta made entirely with fermented faba flour had more homogeneous structure with a delineated edge of the cross-section compared to pasta made with unfermented faba bean flour [52]. Starch granules of faba bean flour were more homogeneous and rounded compared to those of cereal starch [85]. In pasta fortified with more than 30% of fermented faba flour, starch granules were surrounded by protein showing a continuous protein structure. Conversely, when faba flour was used without fermentation, starch granules formed isolated aggregates [51,83].

**Figure 2:**
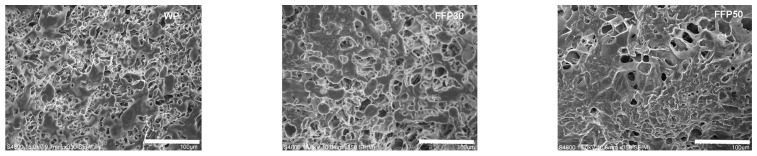
Scanning electron microscopy images (350× magnification, scale bar = 100 µm) of the cross-sectional microstructure of pasta samples cooked at the Optimal Cooking Time (OCT). WP, pasta made with wheat semolina; FFP30 and FFP50, faba bean pasta including 30 or 50% (*w/w*) fermented faba bean flour in replacement of semolina [51].

The microstructure analysis revealed a worsening of the technological properties (e.g., cooking loss, hardness) of pasta occurring when the replacement percentage of semolina with fermented faba bean was higher than 30% [51]. A good aggregation of protein and starch in fermented flour containing pasta was seen up to 30% of wheat semolina replacement. However, when the substitution further increased, micropores appeared [51]. Together with texture weakening, all the nutritional advantages deriving from faba fortification, such as the protein concentration, decreased. Figure 2 reports scanning electron microscopy images of the cross-sectional microstructure of pasta samples fortified with fermented faba bean and cooked at the OCT. 

### 3.3. Cooking Properties of Fortified Products

Overall, the quality of gluten network was recognized as the main factor affecting protein loss in water during pasta cooking. When fortified pasta was produced using fermented instead of raw quinoa flour, a relevant decrease of the OCT was found. Fermentation also caused the decrease of pasta water absorption and led to the increase of cooking loss [80]. Fermentation also decreased the OCT of faba bean pasta compared to that of the controls made with the unfermented faba bean flour or with semolina. The decrease in water retention, directly correlated to the reduction in OCT, was also reported [52]. The same results were observed in another study [51]: the increase of the fortification level (fermented faba bean flour in semolina pasta) led to the decrease of OCT (from 10 min to 6 and 5.5 min at 30% and 50% of replacement, respectively), and the increase of cooking loss. Also in this case, gluten content reduction was reported as the main cause for the different technological behavior. In any case, when fermented faba bean flour was used instead of raw faba bean flour, the effects were further accentuated [51]. 

The partial substitution of wheat flour with pigeon pea affected the OCT of experimental spaghetti. A slight increase of this parameter was found when raw flour was used for fortification, while fermentation led to a decrease of pasta OCT, that resulted similar to that of semolina control. The OCT increase caused by the raw pigeon pea flour fortification is related to the good technological properties of its protein fraction, while proteolysis occurring during extensive fermentation led to the increase of protein loss in water compared to the control pasta [86]. 

Table 2 lists, not exhaustively, the characteristics of the experimental pasta made using fermented non-cereals flour described before.

## 4. Conclusions

To fulfill the request of modern consumers for food with well-balanced nutritional profile and functional properties, the industry is looking for innovation in pasta making. 

As a staple food and a common component of diet, pasta could be considered as a potential vector of dietary fiber, proteins, vitamins, minerals, and functional compounds. The affinity of pasta with baked good products, in which such a revolution is nowadays fully launched, has encouraged the scientific community to set up new biotechnological processes for its fortification. The well-known potential of sourdough fermentation is currently under investigation as a way to provide ingredients that, when included in pasta formulations, could deliver nutritional and functional improvement and peculiar, but still acceptable, sensory characteristics to pasta. Lactic acid bacteria, as the key microorganisms of sourdough fermentation, together with the use of non-wheat flours, represent, until now, an attractive combination to be explored further for the design of the future pasta.

## Figures and Tables

**Table 1:** Non-exhaustive survey of the pasta products including fermentation step of semolina or non-wheat flours mixtures. The main nutritional and functional improvements, and the effects on technological and sensory properties, in comparison to conventional pasta, are reported.

Product	Fermentation Type	Main Results	Reference
Semolina pasta enriched in vitamin B2	Fermentation of semolina (partial and total substitution) with a selected *Lactobacillus plantarum* strain at 42 and 60% *w/w*	Vitamin B2 synthesis.	[42]
Pasta with fermented semolina	Semolina fermentation by *Saccharomyces cerevisiae* and *Lactobacillus alimentarius,* addition to pasta formulation at 30% *w/w*	Low glycemic index.	[54]
		Low cooking loss.	
Semolina pasta with reduced gluten content	Semolina fermentation by a pool of four selected lactic acid bacteria (LAB) at 30% *w/w*	Gluten degradation (*ca.* 83%).	[50]
		No changes in sensory attributes.	
Pasta made with semolina rendered gluten free and pre-gelatinized rice flour	Semolina fermentation by a pool of selected LAB in presence of fungal proteases at 50% *w/w*	Gluten degradation.	[61]
		Increase of the acidic perception.	
Pasta made with parboiled brown rice flour and pre-gelatinized rice flour with sorghum sourdough	Spontaneous fermentation of sorghum, addition to pasta formulation at 15% *w/w*	Low cooking loss, optimal protein network.	[65]

*ca.*: circa.

**Table 2:** Non-exhaustive survey of the pasta products including fermentation step of non-wheat flours mixtures. The main nutritional and functional improvements, and the effects on technological and sensory properties, in comparison to conventional pasta, are reported.

Product	Fermentation Type	Main Results	Reference
Gluten free faba bean pasta	Faba bean flour fermented with selected starters at 100% *w/w*	Increase of protein and resistant starch content.	[52]
		Low beany-flavor perception.	
Pasta fortified with fermented faba bean	Fermentation of faba bean flour with selected starter, addition to pasta formulation at 10, 30, and 50% *w/w*	Increase of protein digestibility and protein quality indexes, reduction of glycemic index degradation of trypsin inhibitors, and condensed tannins.	[51]
Pasta fortified with fermented quinoa	Fermentation of quinoa flour with selected LAB, addition to pasta formulation at 20% *w/w*	Increase of peptides, free amino acids, and total phenol content.	[80]
		Increased of all nutritional indexes and decrease of hydrolysis index.	
		Increase of cooking loss and decrease of water absorption	
Pasta fortified with fermented pigeon pea	Spontaneous fermentation of pigeon pea flour, addition to pasta formulation at 10% *w/w*	Increase of protein quality indexes and chemical scores.	[70,81]
		Sensory acceptability at substitution levels below 10%.	
